# Screening for Pancreatic Adenocarcinoma in BRCA2 Mutation Carriers: Results of a Disease Simulation Model

**DOI:** 10.1016/j.ebiom.2015.11.005

**Published:** 2015-11-04

**Authors:** Pari V. Pandharipande, Alvin Jeon, Curtis R. Heberle, Emily C. Dowling, Chung Yin Kong, Daniel C. Chung, William R. Brugge, Chin Hur

**Affiliations:** a Institute for Technology Assessment, Massachusetts General Hospital, Boston, USA; b Gastroenterology Division, Massachusetts General Hospital, Boston, USA; c Radiology Department, Massachusetts General Hospital, Boston, USA; d Harvard Medical School, Boston, USA

**Keywords:** Pancreatic cancer, BRCA2 mutation, Cancer screening, Simulation disease modeling

## Abstract

**Background:**

BRCA2 mutation carriers are at increased risk for multiple cancers including pancreatic adenocarcinoma (PAC). Our goal was to compare the effectiveness of different PAC screening strategies in BRCA2 mutation carriers, from the standpoint of life expectancy.

**Methods:**

A previously published Markov model of PAC was updated and extended to incorporate key aspects of BRCA2 mutation carrier status, including competing risks of breast- and ovarian-cancer specific mortality. BRCA2 mutation carriers were modeled and analyzed as the primary cohort for the analysis. Additional higher risk BRCA2 cohorts that were stratified according to the number of first-degree relatives (FDRs) with PAC were also analyzed. For each cohort, one-time screening and annual screening were evaluated, with screening starting at age 50 in both strategies. The primary outcome was net gain in life expectancy (LE) compared to no screening. Sensitivity analysis was performed on key model parameters, including surgical mortality and MRI test performance.

**Findings:**

One-time screening at age 50 resulted in a LE gain of 3.9 days for the primary BRCA2 cohort, and a gain of 5.8 days for those with BRCA2 and one FDR. Annual screening resulted in LE loss of 12.9 days for the primary cohort and 1.3 days for BRCA2 carriers with 1 FDR, but resulted in 20.6 days gained for carriers with 2 FDRs and 260 days gained for those with 3 FDRs. For patients with ≥ 3 FDRs, annual screening starting at an earlier age (i.e. 35–40) was optimal.

**Interpretation:**

Among BRCA2 mutation carriers, aggressive screening regimens may be ineffective unless additional indicators of elevated risk (e.g., 2 or more FDRs) are present. More clinical studies are needed to confirm these findings.

**Funding:**

American Cancer Society – New England Division – Ellison Foundation Research Scholar Grant (RSG-15-129-01-CPHPS).

## Highlights

• One-time screening at age 50 resulted in small life expectancy gains for all BRCA2 cohorts.
• Annual screening starting at 50 was effective only for BRCA2 mutation carriers with ≥ 2 FDRs.
• Patients with ≥ 3 FDRs benefitted more from starting annual screening earlier (i.e. age 35–40).

BRCA2 mutation carriers are at higher risk of developing different types of cancers relative to the general population, including pancreatic cancer. Pancreatic cancer screening is not currently advisable for the general population, but could be of benefit to individuals with elevated risk. Using simulation modeling techniques, we projected outcomes for hypothetical cohorts of BRCA2 mutation carriers, with varying numbers of first-degree relatives with a history of pancreatic cancer, that underwent MRI-based pancreatic screening strategies. We found that one-time screening at age 50 in patients with BRCA2 mutations can slightly increase life expectancy. However, more aggressive screening regimens in BRCA2 mutation carriers can be detrimental unless additional risk factors (e.g., two or more affected first-degree relatives) are present.

## 1. Introduction

While the BRCA2 mutation is best known for its associated elevated breast and ovarian cancer risk, it also conveys an increased risk of pancreatic adenocarcinoma (PAC) as the most common genetic alteration found in familial pancreatic cancer. Consequently, some BRCA2 mutation carriers are screened for PAC with imaging. To date, however, consensus guidelines that define how screening should be implemented to benefit mutation carriers – i.e. the frequency and duration of screening – are not available. In a recent statement of the International Cancer of the Pancreas Screening (CAPS) Consortium, most members felt that BRCA2 mutation carriers with ≥ 1 affected first-degree relative, or with two affected relatives (whether or not first degree), should be considered candidates for screening (Canto et al., 2013). However, agreement was not reached on whether mutation carriers without a family history of PAC should be screened.

The lack of consensus largely stems from the limited data available to guide any given PAC screening strategy in BRCA2 mutation carriers. To date, the majority of clinical trials that have been performed to determine the value of PAC screening in high-risk individuals have been small single-arm trials (Canto et al., 2013, Langer et al., 2009, Verna et al., 2010, Al-Sukhni et al., 2012, Ludwig et al., 2011), and none have been specific to BRCA2 mutation carriers. Recently, we used a simulation modeling approach to project potential life expectancy gains for individuals who underwent one-time MRI screening at age 50 and found that gains are possible if a cohort's relative risk for PAC was sufficiently high (Canto et al., 2013). Our findings suggest that screening once at age 50 may be of benefit (Pandharipande et al., 2015). However, our study did not incorporate interval screening for patients who were negative on their first screening study or model patients with specific genetic mutations such as BRCA2.

While some might advocate that the effectiveness of PAC screening in BRCA2 mutation carriers should be evaluated with a clinical trial, such an approach is not practical as a first-line method for addressing this issue, as the scale of the trial (including the number of subjects and required follow-up duration) makes such an approach unfeasible. In such scenarios, a simulation model can be used to gain greater insights into potential benefits of screening in BRCA2 mutation carriers, and also provide data to inform the design of a clinical trial. Equally important, such a model can be probed to identify factors that are likely to drive the success – and failure – of PAC screening efforts in this population. In our current analysis, we substantially modified our previous PAC model to address a population of BRCA2 mutation carriers, and to allow for an MRI screening regimen at regular intervals over many years, reflecting common PAC screening practices in high-risk individuals. Our purpose was to estimate the impact of PAC screening on life expectancy in BRCA2 mutation carriers.

## 2. Methods

### 2.1. Overview of the Model

We previously developed a Markov model to simulate the natural history of PAC in hypothetical populations with varied levels of PAC risk. The model was calibrated to data from the National Cancer Institute's Surveillance, Epidemiology, and End Results (SEER) cancer registry and published literature (Howlader et al., 2013, Key, 2007, Brugge et al., 2004, Tempero et al., 2010; Surveillance, Epidemiology, and End Results (SEER), 2013; Laffan et al., 2008, Canto et al., 2012).

In each simulation, a hypothetical patient cohort begins in a “normal” health state at age 20, and is followed until death or age 100. Patients can progress to PAC via two separate pathways – solid and cystic – which account for 90% vs. 10% of PAC, respectively (Key, 2007, Brugge et al., 2004, Tempero et al., 2010). PACs are further categorized into SEER historic stages. Patients who develop PAC begin with pre-clinical or undetected localized cancer and may progress to more advanced stages. Once diagnosed, patients are subject to stage-dependent mortality rates derived directly from SEER data (Howlader et al., 2013). Probabilities governing transitions between health states were calibrated to data from the SEER registry and published data, as previously described (Pandharipande et al., 2015, Howlader et al., 2013, Key, 2007, Brugge et al., 2004; Surveillance, Epidemiology, and End Results (SEER), 2013; Laffan et al., 2008, Canto et al., 2012).

Screening regimens can be overlaid onto this natural history model, potentially resulting in the earlier detection of PACs. Patients detected in the localized PAC state are treated surgically. In the cystic pathway, screening may also result in detection of precursor lesions (low-risk and high-risk cysts, predominantly intraductal papillary mucinous neoplasms [IPMNs]) (Canto et al., 2012). High-risk cysts prompt surgery, while low-risk cysts undergo annual surveillance for ten years; surgery is prompted in instances of progression. Patients who survive surgical resection of a high-risk cyst return to a baseline (i.e., “normal”) health state, while those who survive surgery for PAC are subject to localized PAC mortality based on SEER estimates (Howlader et al., 2013). While a cure is possible for a subset of these patients, the majority die from their cancer.

SEER data are representative of the U.S. population (Howlader et al., 2013). In order to model subgroups within the population with varying degrees of elevated PAC risk, the baseline risks of developing and dying from PAC were scaled according to a relative risk factor (e.g., 10 × the general population), and model parameter estimates such as transition probabilities governing PAC progression rates were recalibrated to reproduce these higher targets. This approach allowed us to estimate the effect of screening in cohorts with elevated PAC risk.

Full details of model structure and assumptions, parameter estimates, calibration targets and process are available in the published article (Pandharipande et al., 2015). The current analysis included several modifications and extensions to this model, as described below.

### 2.2. Model Incorporation of the BRCA2 Mutation

The current analysis necessitated two principal modifications to our previously validated model. First, in the original model, competing risk of death was determined by age- and gender-specific mortality rates drawn from U.S. life tables for the total population (Arias and United States Life Tables, 2008). However, BRCA2 mutation carriers have an increased risk of breast and ovarian cancers and thus a higher all-cause mortality rate. To account for this, SEER data on age- and cancer-specific relative survival (in the total population) and the age-specific incidence of breast and ovarian cancers (in BRCA2 mutation carriers) were used to adjust the all-cause mortality rates for BRCA2 mutation carriers using a previously applied method (Antoniou et al., 2004, Lee et al., 2008).

Second, the screening component of the model was expanded to incorporate additional screening strategies. In the original model, all patients were evaluated with “one-time” screening at the age of 50. Although formal guidelines are lacking, in clinical practice, patients at high risk for PAC may be screened from ages 50–80, frequently between an annual and triennial basis (Brand et al., 2007). For the current study, we analyzed two screening strategies: one time screening at age 50, and annual screening beginning at age 50 and continuing until age 80.

### 2.3. Elevated Levels of PAC Risk and Additional Key Input Parameters

#### 2.3.1. Risk of Pancreatic Adenocarcinoma (PAC) Compared to the General Population

Estimates of PAC risk in study cohorts of interest were derived from the literature and are included in Table 1. For the primary cohort, a hypothetical cohort of “all-comer” BRCA2 mutation carriers – i.e. those without a pre-specified number of FDRs – were assigned a relative risk of 3.5 × greater than that of the general population (Canto et al., 2013, The Breast Cancer Linkage Consortium, 1999). For higher risk cohorts of BRCA2 mutation carriers with one, two, and three or more FDRs with a history of PAC, each group was assigned relative risks based on the number of affected FDRs as reported in the literature, independent of BRCA2 mutation carrier status. This simplified approach was necessary because data regarding the risk of PAC in patients with both BRCA2 mutations and various numbers of FDRs were not available. Hence, estimates for the increased risk in BRCA2 carriers who also had FDRs may have been low. They were as follows: 4.5 × the general population for patients with one affected FDR, 6.4 × for patients with two affected FDRs, and 32.0 × for patients with three or more FDRs (Canto et al., 2013, Grover and Syngal, 2010). All-cause mortality rates for these patients still reflected the BRCA2-adjusted mortality rates.

#### 2.3.2. MRI Test Performance

Data from six single-arm patient studies that utilized PAC screening with first-line MRI (including MR cholangiopancreatography, and with concurrent or follow-up EUS in most cases with positive MRI results) were used to compute the sensitivity and specificity of parameters used in the model (overall sensitivity = 56%, overall specificity = 97% (Table 1)) (Langer et al., 2009, Verna et al., 2010, Al-Sukhni et al., 2012, Ludwig et al., 2011, Canto et al., 2012, Vasen et al., 2011). We classified cases from these studies into true-positive, false-negative, true-negative, and false-positive categories to derive these estimates using uniform criteria, as previously described (Pandharipande et al., 2015). We assumed independent results between screens.

We applied MRI test performance characteristics in the model as follows (Pandharipande et al., 2015). In the solid pathway, we assumed precursor lesions (pancreatic intraepithelial neoplasias) could not be detected. Therefore, in the solid pathway, false-negative cases were those in which patients localized PAC were misclassified as healthy, and false-positive cases were those in which healthy patients were misclassified as having PAC. In the cystic pathway, detection of low-risk and high-risk cysts (precursor lesions) was possible in our model. The proportion of patients with cysts who ultimately underwent surgery in a large screening trial was used as a proxy to determine the proportion of patients with high-risk (vs. low-risk) cysts (Pandharipande et al., 2015, Canto et al., 2012). In the cystic pathway, false-negative cases were those in which a low-risk cyst, high-risk cyst, or localized PAC was missed on screening, while false-positive cases were those in which a low-risk cyst was misclassified as high-risk (or as PAC). We did not incorporate the possibility of misdiagnosing a healthy patient as having a cyst or a cyst-derived PAC. The same test performance characteristics were used to determine the probability of each type of error in classification.

#### 2.3.3. Surgical Mortality

Patients with high-risk cystic lesions or localized PAC underwent surgery and were subject to surgical mortality risk. The surgical mortality rate may be impacted by factors such as patient age, performance status, and institution (Pellegrini et al., 1989, Crist et al., 1987, Fernandez-del Castillo et al., 2012, Finks et al., 2011, Winter et al., 2006, Reames et al., 2014). In our current model, we used a surgical mortality rate of 1%, a rate on the lower end of the published rates ranging from 1 to 6%, because asymptomatic patients tend to be healthier than symptomatic patients diagnosed with cancer, and are therefore more likely to survive surgery (Table 1) (Pellegrini et al., 1989, Crist et al., 1987, Fernandez-del Castillo et al., 2012, Finks et al., 2011, Winter et al., 2006, Reames et al., 2014).

### 2.4. Sensitivity Analyses

In sensitivity analysis, we evaluated additional screening strategies (in which the starting age of screening and screening intervals were further varied) to determine effects on model results. In addition, we performed extensive one-way deterministic analyses to evaluate the stability of our results to varied estimates of MRI sensitivity, MRI specificity, and surgical mortality.

### 2.5. Role of the Funding Source

The model development and analysis, and interpretation of the model results – as well as manuscript writing, preparation, and the decision to submit the manuscript – were at the sole discretion of the authors and not the Sponsor.

## 3. Results

Changes in life expectancy associated with the two screening strategies (one-time and annual screening) across all study cohorts are included in Table 2. One-time screening at age 50 for the primary cohort resulted in a LE gain of 3.9 days. One-time screening in cohorts with affected FDRs had greater returns: 5.8 days of life gained for BRCA2 carriers with one FDR, 9.1 days with two FDRs, and 31.5 days with three or more FDRs. Annual screening from ages 50–80 resulted in a LE loss of 12.9 days for the primary cohort as well as a loss of 1.3 days for BRCA2 carriers with one affected FDR. However, annual screening resulted in LE gains of 20.6 days with two FDRs and 260.0 days with three or more FDRs.

**Fig. 1:**
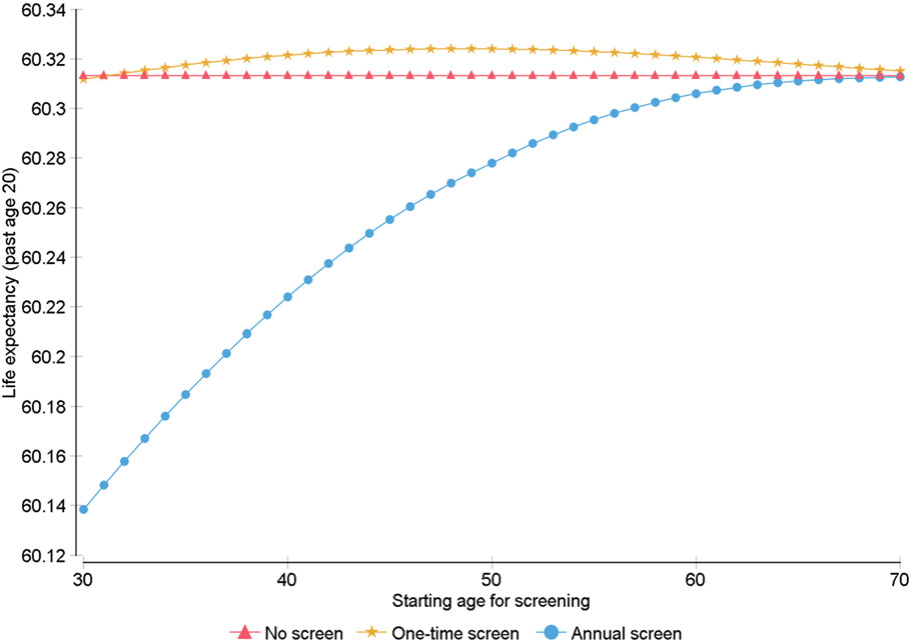
Sensitivity analysis for starting age for screening in primary cohort BRCA2 carriers. Starting age was varied over a range of 30–70, and for each strategy, life expectancy past age 20 was measured.

**Fig. 2:**
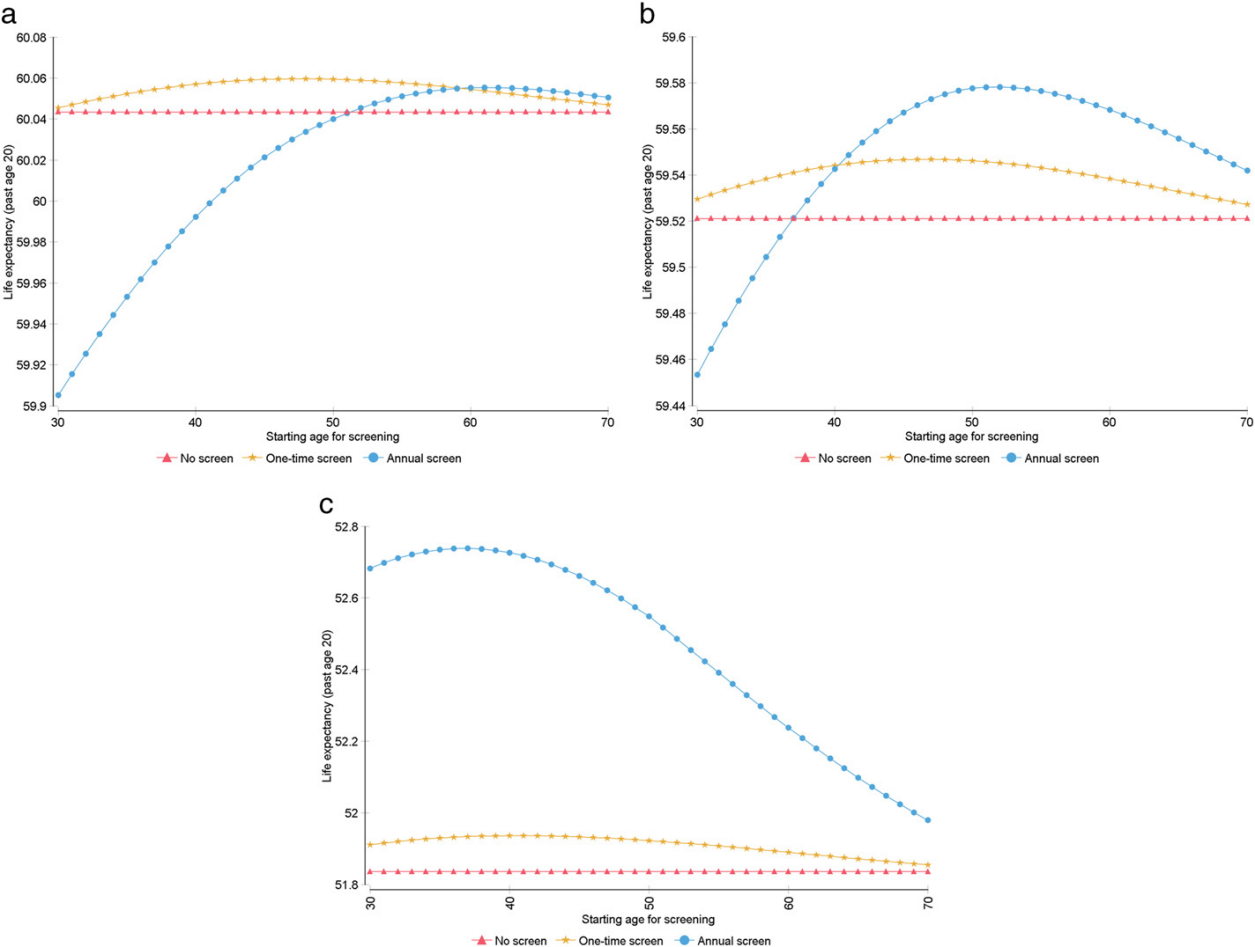
Sensitivity analysis for starting age for screening in BRCA2 carriers with one (A), two (B), and three or more (C) first degree relatives. Starting age was varied over a range of 30–70, and for each strategy, life expectancy past age 20 was measured.

Sensitivity analysis shows changes in LE gains when varying starting age for screening, over a range of 30–70, for each strategy. For the primary cohort, LE gains persisted for starting ages above 32 and peaked when applying a strategy of one-time screening at age 49 (Fig. 1). For cohorts with affected FDRs, the results are as follows: With one affected FDR, LE gains persisted for all starting ages and peaked with one-time screening at age 48 (Fig. 2a). With two FDRs, LE gains peaked with annual screening starting at age 52 (Fig. 2b). With three or more FDRs, LE gains peaked with annual screening starting at age 37 (Fig. 2c).

**Fig. 3:**
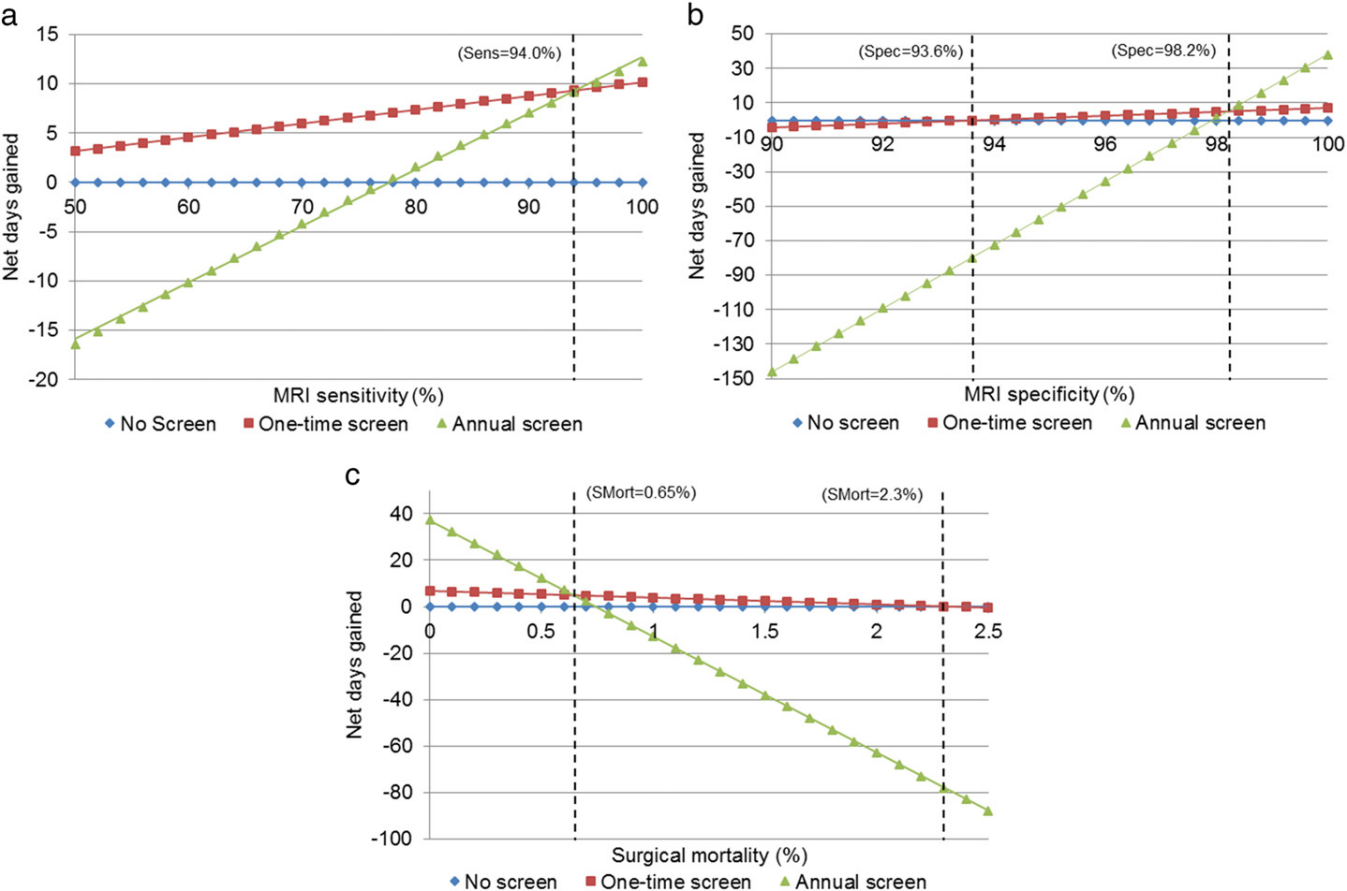
Sensitivity analysis for MRI sensitivity (A), MRI specificity (B), and surgical mortality (C). Sensitivity (Sens) was varied over a range of 25–100% (50–100% graphed), specificity (Spec) over a range of 50–100% (90–100% graphed), and surgical mortality (SMort) over a range of 0–10% (0.0–2.5% graphed). For each strategy, net days gained by screening for PAC in individuals with BRCA2 mutations were measured.

Results were also sensitive to changes in MRI sensitivity. For the primary cohort, the values tested ranged from 25 to 100% (Fig. 3a, 50–100% graphed). The threshold value where one-time screening was no longer effective (i.e. resulted in LE loss) was when sensitivity was less than 27.7%. The threshold value where one-time screening was no longer superior to annual screening was when sensitivity was greater than 94.0%. For cohorts with affected FDRs, thresholds at which point one-time screening at age 50 was no longer effective were: 23.2%, 18.8%, and 15.8% for one, two, and three or more FDRs, respectively. Thresholds where one-time screening was no longer superior to annual screening were: 67.2%, 43.6%, and 16.4% for one, two, and three or more FDRs, respectively.

Results were also sensitive to changes in MRI specificity. For the primary cohort, the values tested ranged from 50 to 100% (Fig. 3b, 90–100% graphed). The threshold value where one-time screening was no longer effective was when specificity was less than 93.6%. The threshold value where one-time screening was no longer superior to annual screening was when specificity was greater than 98.2%. For the cohorts with affected FDRs, thresholds at which point one-time screening at age 50 was no longer effective were: 91.8%, 88.6%, and 53.0% for one, two, and three or more FDRs, respectively. Thresholds where one-time screening was no longer superior to annual screening were: 97.6%, 96.6%, and 78.2% for one, two, and three or more FDRs, respectively.

Results were also sensitive to changes in surgical mortality. For the primary cohort, the values tested ranged from 0 to 10% (Fig. 3c, 0.0–2.5% graphed). One-time screening was no longer effective was when surgical mortality rates were greater than 2.3%. The threshold value for when one-time screening was no longer superior to annual screening was when surgical mortality was below 0.6%. For the cohorts with affected FDRs, thresholds at which point one-time screening at age 50 was no longer effective were: 3.0% and 4.2% for one and two FDRs, respectively, and the threshold was above 10% for three or more FDRs. Thresholds where one-time screening was no longer superior to annual screening were: 0.8%, 1.2%, and 8.1% for one, two and three or more FDRs, respectively.

### 3.1. Screening Interval

A sensitivity analysis on screening interval showed that for the primary cohort, screening less frequently than every 3.9 years led to a gain in LE. Similarly, screening BRCA2 mutation carriers with one FDR less often than 1.4 years led to a net LE gain. For the higher risk groups with either two FDRs or three or more FDRs, screening less frequently than annually resulted in diminished LE gain.

### 3.2. Two-way Sensitivity Analysis

**Fig. 4:**
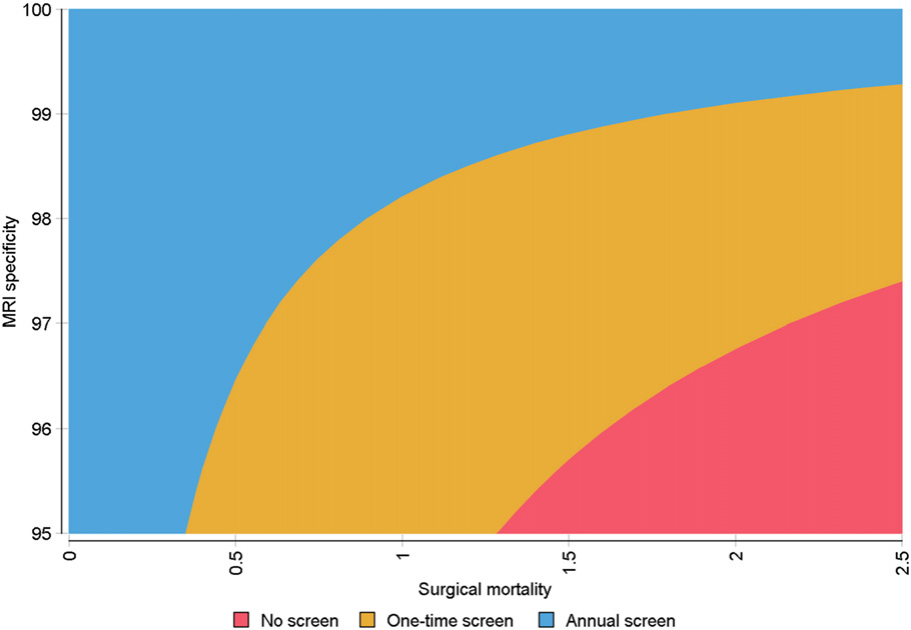
Two-way sensitivity analysis on effectiveness of screening in primary cohort BRCA2 carriers. Surgical mortality and MRI specificity were varied over ranges of 0–10% (0–2.5% graphed) and 50–100% (95–100% graphed), respectively.

We also performed a two-way deterministic sensitivity analysis of surgical mortality rate and MRI specificity. The interaction of the two parameters is presented in Fig. 4. Generally, the combination of lower surgical mortality rates and higher MRI specificity values leads to annual screening being superior.

## 4. Discussion

We found that one-time screening of BRCA2 mutation carriers can increase life expectancy. Despite heightened PAC risk in this cohort, more aggressive screening at regular intervals may result in diminished life expectancy. This finding can be attributed to a phenomenon that is increasingly recognized in cancer screening initiatives: an increased number of false-positive results, which can occur when healthy patients are screened repeatedly, can lead to more harm than benefit. This is particularly important in the setting of PAC, given the relatively high morbidity and mortality risks of pancreatic surgery. When we evaluated subsets of BRCA2 mutation carriers with varying numbers of FDRs, we found that only those with multiple FDRs (two or more) would benefit from annual screening. Conversely, high-risk BRCA2 mutation carriers with three or more FDRs might benefit from an even earlier starting age for screening. In this setting, PAC risk is high enough that the benefits of increased screening frequency outweigh the harms. In addition to the burden of false-positive results, the benefit of screening is significantly limited, even in high risk individuals, by the poor efficacy of PAC treatment, an area of medicine in dire need of improvement. Five year survival for patients with PAC diagnosed at an early stage and who undergo successful resection with clear surgical margins (R0 resection) remains low at < 20% (Wagner et al., 2004). Even within this select group, over 80% of PAC tumors recur with metastatic disease, as many of these patients already have micrometastases at diagnosis that are not detectable by current imaging modalities (Garrido-Laguna and Hidalgo, 2015). Promising new adjuvant and neoadjuvant therapeutic regimens may improve survival for early stage PAC patients(Neoptolemos et al., 2010, Oettle et al., 2007) and could significantly alter the calculus for screening benefit in the near future.

Previous smaller studies have found that screening in high-risk cohorts may be able to detect preinvasive pancreatic lesions amenable to interventions such as surgery (Verna et al., 2010, Canto et al., 2012), although the relatively low number of positive findings in screening studies and a lack of evidence that early identification will improve cancer survival or outcomes has tempered enthusiasm (Langer et al., 2009). A 2007 consensus conference proposed that screening for PAC be restricted to individuals with greater than ten-fold increased risk (Brand et al., 2007). Additionally, the National Comprehensive Cancer Network guidelines do not currently recommend pancreatic screening for patients with BRCA mutations. The lack of guidelines and data to inform clinical practice was the primary motivation for our analysis.

A strength of our analysis was that our model was calibrated to NCI SEER data, making many aspects of the natural history generalizable to the US population. An additional strength was that our model incorporated the risks of death from the key cancers associated with BRCA2 mutation, breast and ovarian, into all-cause mortality rates, thereby providing more accurate estimates for life expectancies within this unique cohort of patients.

Our study had limitations. Potential interactions between BRCA2 mutation carrier status and the number of FDRs afflicted with PAC were not available in the literature and therefore simplifying assumptions regarding their independence were made. Although our model inputs were based on the published literature, in some cases limited data were available, creating uncertainty; to address this, extensive sensitivity analyses were performed to assess the robustness of the findings. In addition, MRI screening performance continues to evolve and novel imaging technologies are a focus of much research; improvements to a screening modality in which both sensitivity and specificity increase could affect our conclusions. Moreover, we did not address EUS as a first-line imaging strategy for PAC screening; while our sensitivity analysis results provide insight into potential benefits (or losses) associated with a spectrum of possible test performance characteristics, future studies that compare MRI and EUS, and which further examine their complementary capabilities for lesion detection, are warranted. Future clinical studies are needed to confirm our estimates of the impact of screening on PAC survival in BRCA2 cohorts.

Although our study was geared towards guiding PAC screening in BRCA2 mutation carriers, our findings suggest that screening may be effective in other patients or cohorts with genetic syndromes such as Familial Atypical Multiple Mole Melanoma Syndrome and Peutz–Jeghers Syndrome, which are less prevalent but are associated with extremely high risks of PAC; the optimal screening regimen, however, would need to be analyzed in separate analyses.

In conclusion, we found that in BRCA2 mutation carriers, one-time MRI-based PAC screening at age 50 can be effective and increase life expectancy, albeit with marginal benefit. More aggressive screening at regular intervals may be harmful, as the number of false positives accumulates with repeat examinations, and the burden of the resulting surgical interventions may outweigh the benefit of cancer mortality reduction. If the risk of PAC is very high, such as in patients with multiple first degree relatives with PAC, annual MRI screening (starting at age 50 for two FDRs and age 35–40 for three or more FDRs) may be optimal. Future clinical studies are needed to confirm our findings.

## Contributors

“PVP, AJ, CRH, ECD, CYK, DCC, WB, CH contributed to study design. PVP, AJ, and CRH contributed to data collection. PVP, AJ, CRH, DCC, WB, and CH contributed to data analysis. All authors contributed to the interpretation of the data and all authors critically reviewed the manuscript and approved the final version for submission.”

## Declaration of Interests

PVP receives research funding from the Medical Imaging and Technology Alliance for unrelated work. WB is a consultant for Boston Scientific and receives research funding from Lustgarten, Celgene, and RedPath for unrelated work. All other authors declare no competing interests.

## Figures and Tables

**Table 1:** Relative risk of PAC in BRCA2 cohorts, surgical mortality, and screening performance.

Parameter	Value	References
Relative risk of PAC		
Primary cohort^a^	3.5	Canto et al. (2013) and The Breast Cancer Linkage Consortium (1999))
# of FDRs^b^		
1	4.5	Canto et al. (2013) and Grover and Syngal (2010)
2	6.4	Canto et al. (2013) and Grover and Syngal (2010)
≥ 3	32.0	Canto et al. (2013) and Grover and Syngal (2010)
Surgical mortality rate		
Surgical mortality	1%^c^	Pellegrini et al. (1989), Crist et al. (1987), Fernandez-del Castillo et al. (2012), Finks et al. (2011), Winter et al. (2006) and Reames et al. (2014)
Test performance characteristics for MRI screening strategy		
Sensitivity	56% (5/9)	Langer et al. (2009), Verna et al. (2010), Al-Sukhni et al. (2012), Ludwig et al. (2011), Canto et al. (2012) and Vasen et al. (2011)
Specificity	97% (739/760)	Langer et al. (2009), Verna et al. (2010), Al-Sukhni et al. (2012), Ludwig et al. (2011), Canto et al. (2012) and Vasen et al. (2011)

a BRCA2 mutation carriers without a specific number of FDRs (i.e. “all-comers”).

b Relative risks for BRCA2 mutation carriers with affected FDRs.

c Range = 1–6%; 1% estimate weighted towards more favorable outcomes in screen-detected patients; range from 0 to 10% tested in sensitivity analysis.

**Table 2:** Net life expectancy (days) gained by screening for PAC in individuals with BRCA2 mutations.

	BRCA2 mutation carriers		
Strategy	No screening	One-time screening: age 50	Annual screening: ages 50–80
Primary cohort^a^	–	**3.9**^b^	− 12.9
# of FDRs			
1	–	**5.8**^b^	− 1.3
2	–	9.1	**20.6**^b^
≥ 3	–	31.5	**260.0**^b^

a BRCA2 mutation carriers without a specific number of FDRs (i.e. “all-comers”).

b The numbers **bolded** represent the “optimal” strategy for the cohort as determined by most net gain in life expectancy (days gained).
